# Association of pre-operative depressive and anxiety symptoms with five-year survival of glioma and meningioma patients: a prospective cohort study

**DOI:** 10.18632/oncotarget.15743

**Published:** 2017-02-25

**Authors:** Adomas Bunevicius, Vytenis Pranas Deltuva, Arimantas Tamasauskas

**Affiliations:** ^1^ Neuroscience Institute, Lithuanian University of Health Sciences, Kaunas, Lithuania

**Keywords:** glioma, meningioma, depression, anxiety, prognosis

## Abstract

**Background:**

Mental symptoms are common and associated with worse health status of brain tumor patients. We evaluated the association of pre-operative depressive and anxiety symptoms with 5-year mortality of glioma and meningioma patients.

**Methods:**

One-hundred and fifty-two patients (mean age 56.9±14.7 years, 69% women) were evaluated for functional status (Barthel index), and depressive and anxiety symptom severity (Hospital Anxiety and Depression scale or HADS). Patients were categorized as having mild, moderate or severe depressive/anxiety symptoms if they scored ≤7, 8-10 or ≥11 on the HADS, respectively. Information pertaining to histological diagnosis, extent of resection and adjuvant therapies were obtained from medical records. Follow-up continued through November, 2015.

**Results:**

Forty-three patients were diagnosed with high-grade glioma, 20 with low-grade glioma and 89 with meningioma. Moderate to severe depressive and anxiety symptoms were diagnosed in 28% and 36% of patients, respectively. In meningioma patients, survival was the shortest in patients with severe depressive symptoms (40.32±7.92 months) followed by patients with moderate (46.66±6.05 months) and mild (55.68±1.77 months) depressive symptoms (Log-Rank = 6.211, *p* = 0.045). After adjusting for patients’ age, gender, functional status, extent of resection, history of depression, and tumor location, laterality and grade, severe depressive symptoms were associated with increased 5-year mortality risk of meningioma patients (HR = 7.083 [95%CI: 1.755–28.588], *p* = 0.006). Depressive and anxiety symptoms were not associated with mortality of glioma patients

**Conclusions:**

Depressive and anxiety symptoms are common in glioma and meningioma patients. Pre-operative depressive symptoms are associated with shorter survival of meningioma patients independently from clinical prognostic indicators.

## INTRODUCTION

Primary brain tumors are rare diseases with annual age-adjusted incidence rate of 28.57 per 100,000 population [1]. Glioblastoma is associated with progressive clinical course and overall survival rarely exceeds 12 months [1, 2]. Low-grade gliomas can be controlled for a prolonged period of time [3]; however, malignant transformation is common. Meningioma can be cured following gross total surgical resection, but it can be difficult to achieve for deep-seated skull base tumors [4].

Psychiatric disorders and symptoms are common complications of brain tumors [5, 6]. Fifteen percent of glioma patients meet diagnostic criteria for depressive disorder and one third of patients suffer from subclinical depressive symptoms [6]. Depression is also the most common psychiatric symptom of meningioma patients [7] affecting more than 20% of patients [8]. Depression is associated with greater functional disability [9], impaired quality of life [10] and worse cognitive functioning [6] of brain tumor patients. The association of depression with survival of brain tumor patients remains unclear. In high grade glioma patients, two previous studies found that depressive disorder predicted shorter survival [11, 12], while another prospective study did not find an association between depressive symptoms patients prognosis [13]. Depressive symptoms were also associated with a shorter survival of patients with low-grade gliomas, but not benign brain tumors [13]. Therefore, studies investigating prognostic value of depression in brain tumor patients are needed.

Anxiety is another common neuropsychiatric complication in brain tumor patients with point prevalence rates of anxiety symptoms ranging from 30% [14] [15] to 63% [16]. Anxiety symptoms are associated with worse quality of life and worse cognitive functioning of glioma and meningioma patients [8, 10, 15]. However, there are no studies investigating the prognostic value of anxiety in brain tumor patients. The association of anxiety symptoms with survival in non-CNS cancer patients remains inconclusive. For example, anxiety symptoms predicted shorter survival of terminally ill cancer patients [17]. However, others did not find an association between anxiety and survival of ovarian cancer patients [18] or reported that lower anxiety levels were associated with shorter survival of breast cancer patients [19].

Brain tumor histological diagnosis and grade, patient’s age and functional status are the most important clinical prognostic indicators of brain tumor patients [20]. Identification of modifiable prognostic indicators, such as mental symptoms, can be important towards improving prognosis of brain tumor patients. Therefore, the aim of the study was to evaluate the association of pre-operative depressive and anxiety symptoms with 5-year mortality of glioma and meningioma patients.

## MATERIALS AND METHODS

### Patients

In a period from March, 2010 until September, 2011 consecutive adult patients admitted for brain tumor surgery at the Department of Neurosurgery of the Hospital of Lithuanian University of Health Sciences, Kaunas, Lithuania were invited to participate in this prospective observational cohort study. Patients who were unable to comprehend the study tasks due to inability to speak Lithuanian or severe neurological deficits were excluded.

During the study period 232 patients were evaluated for depressive and anxiety symptom severity. However, 78 (34%) patients were excluded because they were diagnosed with other histological types of brain tumors than glioma and meningioma. Therefore, our final sample included 152 patients (69% women and 31% men; mean age 56.89 ± 14.69 years) diagnosed with high-grade glioma (*n* = 43), low-grade glioma (*n* = 20) and meningioma (*n* = 89).

### Study design

The study and its consent procedures were approved by the Ethics Committee for Biomedical Research at the Lithuanian University of Health Sciences, Kaunas, Lithuania. Each patient gave written informed consent prior to inclusion in the study.

After admission and before elective surgery patients were interviewed for socio-demographic characteristics (age, gender, marital status and education) and clinical characteristics (histories of depressive disorder, current use of psychotropic medication, brain tumor type [primary or recurrent brain tumor], tumor side and location). During the same visit all patients were evaluated for functional status (Barthel index or BI), and were asked to complete the Hospital Anxiety and Depression Scale (HADS) as a paper-and pencil questionnaire for evaluation of depressive and anxiety symptom severity. All evaluations were performed by a trained study researcher. Extent of tumor removal was classified as gross total, subtotal or biopsy as noted by the operating neurosurgeon in the operative report. The final histological diagnoses and World Health Organization (WHO) tumor grade [21] were noted by reviewing pathology reports. All patients received adjuvant treatment according to the existing guidelines. Information on adjuvant therapies were obtained from the medical records. Data regarding 5-year mortality were collected from the national mortality registry.

### Depressive and anxiety symptom assessment

The HADS is comprised of two 7-item subscales of anxiety (HADS-Anxiety) and depression (HADS-Depression) that are designed to measure respective symptom severity on a four-point Likert-type scale, with scores on each item ranging from 0 to 3 [22]. Total score on the HADS-Anxiety and HADS-Depression ranges from 0 to 21 with greater score indicating greater symptom severity. Patients were categorized as having mild, moderate or severe depressive or anxiety symptoms if they scored 7 or less, between 8 and 10 and 11 or more, respectively, on the HADS-Depression and HADS-Anxiety, respectively.

The HADS is the most commonly used self-rating instrument for research purposes in neuro-oncology setting and was recommended for initial depression screening in brain tumor patients [6]. Lithuanian translation of the HADS is well-validated for depression [23] and anxiety screening [24] and is commonly used in brain tumor patients [25, 26].

### Mortality

Data on five-year all-cause mortality were collected from the national death registry by using the Lithuanian equivalent of social security number. Deaths that occurred between the study entry date and November, 2015 were considered for the study. No patients were lost to follow-up. Causes of death were coded according to the International Statistical Classification of Diseases and Related Health Problems, Tenth Revision, Australian Modification (ICD-10-AM) [27].

### Statistical analyses

Differences between groups were tested using the Chi-squared test for categorical variables, and Mann-Whitney U test, Kruskal-Wallis test and ANOVA analysis for continuous variables. The Kaplan-Meier survival analysis with log-rank test was used to assess statistical significance of difference in the length of survival between the subgroups of patients stratified by depressive symptom severity (HADS-D score of ≤7 vs. 8-10 vs. ≥11) and anxiety symptom severity (HADS-A score of ≤7 vs. 8-10 vs. ≥11). Cox regression analyses (enter) were performed to evaluate the association of severe depressive and anxiety symptom severity with 5 year mortality adjusting for patients’ age ( < 50 vs. ≥50 years), gender (male vs. female), tumor type (primary vs. recurrent), functional status (BI score 100 vs. < 100), extent of resection (complete vs. incomplete or biopsy), adjuvant treatment (yes vs. no), histories of depression (yes vs. no), and tumor laterality, location and grade. Survival analyses were performed separately for patients with high-grade gliomas, low-grade gliomas and meningiomas. All statistical analyses were performed using the SPSS statistical software, version 10 (SPSS, Inc., Chicago, IL).

## RESULTS

Baseline demographic and clinical characteristics of the study cohort are presented in Table 1. The majority of the study patients were 50 years old or older (69%), lived with a partner (75%), were high-school graduates (38%), had BI score of 100 (73%), underwent initial brain tumor surgery (84%) and gross total resection (86%). Low grade glioma patients were the youngest (*p* < 0.001), while functional status was the lowest in high-grade glioma patients (*p* = 0.009) relative to patients with other histological diagnoses. In high-grade glioma patients there were 38 (88%) patients with WHO grade IV glioma and 5 (12%) patients with WHO grade III glioma. In low grade-glioma patients, 7 (37%) patients were diagnosed with WHO grade I and 12 (63%) patients with WHO grade II gliomas. There were 75 (84%) WHO grade I, 12 (14%) WHO grade II and 2 (2%) WHO grade III meningiomas. Ten (7%) of patients had histories of depressive disorder and 14 (9%) patients were currently taking psychotropic medications (benzodiazepines or antidepressants). Among patients with mild to moderate depressive and anxiety symptom severity 2 (14%) and 5 (8%) patients, respectively, were currently taking psychotropic medication. Depression histories and psychotropic medication use were not different between brain tumor histological diagnoses (*p* = 0.11 and *p* = 0.35, respectively). Tumor side and location were not associated with HADS-A and HADS-D scores (Table 2).

**Table 1:** Baseline demographic and clinical characteristics of the study patients

Characteristic	All patients	High-grade glioma, n=43	Low grade glioma, n=20	Meningioma, n=89	Significance
Age Mean ± SD, Median [IQR] <50 years, n (%) ≥50 years, n (%)	56.9±14.7, 59 [21] 47 (31%) 105 (69%)	58.9±13.8 59 [19] 9 (21%) 34 (79%)	39.0±13.4 37 [18] 16 (80%) 4 (30%)	59.9±12.5 61 [19] 22 (25%) 67 (75%)	X^2^=27.24, p<0.001 X^2^=26.17, p<0.001
Gender, n (%) Men Women	47 (31%) 105 (69%)	18 (42%) 25 (58%)	11 (55%) 9 (45%)	18 (20%) 71 (80%)	X^2^=12.61, p=0.002
Marital status, n (%) With partner Single	108 (75%) 37 (25%)	32 (80%) 8 (20%)	14 (78%) 4 (22%)	62 (71%) 25 (29%)	X^2^=2.76, p=0.60
Education, n (%) Not graduated from high school Graduated from high school Some university education Graduated from university	19 (13%) 55 (38%) 40 (28%) 28 (19%)	7 (18%) 13 (33%) 13 (33%) 7 (18%)	1 (6%) 6 (33%) 7 (39%) 4 (22%)	11 (13%) 36 (42%) 20 (23%) 17 (20%)	X^2^=10.32, p=0.41
History of depression	10 (7%)	1 (2%)	0	9 (10%)	X^2^=4.384; p=0.11
Current use of psychotropic medication	14 (9%)	2 (5%)	3 (16%)	9 (10%)	X^2^=2.126; p=0.35
Barthel index, n (%) Score <100	41 (27%)	18 (43%)	1 (6%)	22 (26%)	X^2^=9.33, p=0.009
Recurrent tumor, n (%)	24 (16%)	10 (25%)	2 (10%)	12 (14%)	X^2^=2.98, p=0.2
Extent of resection, n (%) Complete Incomplete or biopsy	130 (86%) 22 (14%)	35 (88%) 5 (12%)	17 (85%) 3 (15%)	78 (93%) 6 (7%)	X^2^=1.62, p=0.4
Adjuvant therapy, n (%) Yes Radiotherapy Chemotherapy	47 (31%) 44 (29%) 19 (13%)	34 (79%) 33 (77%) 16 (37%)	7 (35%) 7 (35%) 1 (5%)	6 (7%) 4 (5%) 2 (2%)	X^2^=71.19, p<0.001 X^2^=73.99, p<0.001 X^2^=33.59, p<0.001
HADS-Depression score, n (%) ≤ 7 8 – 10 ≥ 11	110 (73%) 22 (15%) 19 (12%)	27 (63%) 7 (16%) 9 (21%)	17 (90%) 1 (5%) 1 (5%)	66 (74%) 14 (16%) 9 (10%)	X^2^ = 6.259; p=0.18
HADS-Anxiety score, n (%) ≤ 7 8 – 10 ≥ 11	96 (64%) 32 (21%) 23 (15%)	29 (67%) 8 (19%) 6 (14%)	12 (63%) 5 (26%) 2 (11%)	55 (62%) 19 (21%) 15 (17%)	X^2^ = 0.994; p=0.91

HADS, Hospital Anxiety and Depression Scale; IQR, interquartile range

**Table 2:** The association of depression and anxiety symptom severity with tumor location and side (mean ± SD)

		Hospital Anxiety and Depression scale	
	Number of patients	Depression	Anxiety
**Tumor location**			
Frontal	61	4.52±4.13	5.74±4.05
Parietal	24	6.33±5.08	7.17±4.58
Occipital	8	5.13±3.314	5.75±3.81
Temporal	40	4.83±4.02	6.18±4.16
Posterior fossae	7	3.09±4.85	5.73±4.86
Other	11	5.57±4.58	6.43±6.05
F; p		1.055; 0.388	0.416; 0.837
**Tumor side**			
Right hemisphere	64	5.48±4.48	6.58±4.33
Left hemisphere	69	4.28±4.04	5.67±4.12
Bilateral	18	4.71±4.62	5.59±4.14
F; p		1.194; 0.314	2.440; 0.067

**Figure 1:**
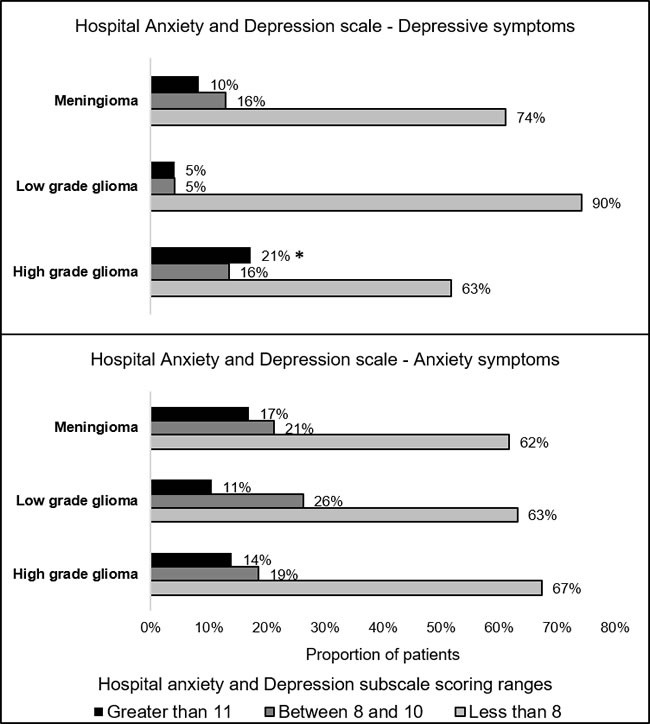
Prevalence of anxiety and depression symptoms *Proportion greater relative to patients with low-grade glioma and meningioma (*X*^2^ = 3.896, *p* = 0.048).

In the total sample, 22 (15%) patients and 19 (13%) patients scored from 8 to 10 and ≥11 on the HADS-Depression subscale, respectively. Thirty-two (21%) patients and 23 (15%) patients scored from 8 to 10 and ≥11 on the HADS-Anxiety subscale, respectively. The proportion of patients scoring ≥ 11 on the HADS-D was greater in high-grade glioma patients (21%) relative to meningioma and low grade glioma patients (9%) (Pearson *X*2 = 3.896, *p* = 0.048) (Table 1 and Figure 1). The proportion of patients with HADS-A scores of ≥11 was not different as a function of histological diagnosis (*p* = 0.783). Median HADS-A score was greater in patients with histories of depression relative to patients without depression histories (10 [12] vs. 6 [6], *p* = 0.41). HADS-D score was not different as a function of depression history (*p* = 0.14). HADS-A and HADS-D scores were similar between patients who were taking psychotropic medication versus patients who did not (*p* = 0.76 and *p* = 0.81, respectively).

**Figure 2:**
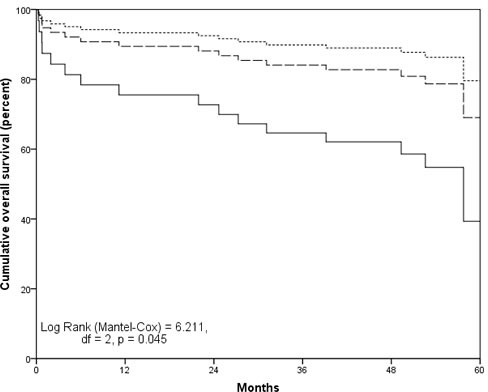
Five-year survival as a function of preoperative depressive symptoms in patients with meningioma Hospital Anxiety and Depression scale Depression subscale score ≤7 (dotted line), from 8 to 10 (dashed line) and ≥11 (solid line).

Survival was the shortest in high-grade glioma patients (13.55 ± 13.86 months), relative to low-grade glioma patients (48.51 ± 15.81 months) and meningioma patients (46.74 ± 15.56 months; Kruskall-Wallis test *p* < 0.001). Forty (93%) high-grade glioma patients, 3 (16%) low grade glioma patients and 17 (19%) meningioma patients died during the five-year follow-up period. In high-grade glioma patients 39 patients died due to brain tumor progression and one patient died due to hemorrhagic stroke. In low-grade glioma patients causes of death were tumor progression (*n* = 2) and ischemic stroke (*n* = 1). In meningioma patients, causes of death were meningioma progression (*n* = 8), coronary artery disease (*n* = 4), cancers of other site (n = 2), cerebrovascular disease (*n* = 1), pneumonia (*n* = 1) and obstructive pulmonary disease (*n* = 1). The distribution of causes of death was not different as a function of depression and anxiety symptom severity (*p*–values≥0.16). Kaplan-Meier analyses demonstrated that greater preoperative depressive symptom severity was associated with shorter overall survival of meningioma patients. Overall survival time was the shortest in meningioma patients with severe depressive symptoms (40.32 ± 7.92 months) followed by patients with moderate depressive symptom severity (46.66 ± 6.05 months) and by patients with low depressive symptom severity (55.68±1.77; Log-Rank = 6.211, df = 2, *p* = 0.045) (Figure 2 and Table 3). Anxiety symptoms were not associated with survival of meningioma patients (*p* = 0.74). Preoperative depressive and anxiety symptoms were not associated with overall survival of high-grade glioma patients (*p* = 0.55 and p = 0.88, respectively) and low-grade glioma patients (*p* = 0.12).

**Table 3:** The association of preoperative depressive and anxiety symptom severity with overall survival (in months) of primary brain tumor patients

	Meningioma	Low-grade glioma	High-grade glioma
**Depressive symptoms: Hospital Anxiety and Depression Scale – Depression subscale**			
Mild (score ≤ 7) Moderate (score from 8 to 10) Severe (score ≥ 11)	55.68±1.77 46.66±6.05 40.32±7.92	55.98±3.17 7.6 54.23	12.19±2.75 17.62±6.90 15.41±3.86
Log-rank test; df; p-value	6.211; 2; 0.045	-	0.208; 2; 0.547
Adjusted analyses (p-value) A	**0.006**	0.971	0.386
**Anxiety symptoms: Hospital Anxiety and Depression Scale – Anxiety subscale**			
Mild (score ≤ 7) Moderate (score from 8 to 10) Severe (score ≥ 11)	51.63±2.16 51.52±5.58 54.74±3.80	48.04±4.50 55.61±4.62 29.98±22.38	12.98±2.68 16.77±6.38 12.65±2.34
Log-rank test; df; p-value	0.616; 2; 0.735	4.210; 2; 0.12	0.251; 2; 0.88
Adjusted analyses ^A^	0.779	0.941	0.809

^A^ Adjusted for patients’ age, gender, tumor type, functional status, extent of resection, and adjuvant treatment, histories of depression, and tumor laterality, location and grade.

In bold, *p* value <0.05

In univariate Cox regression analyses, meningioma patients with severe depressive symptoms (HADS-D score ≥ 11) before surgery were at increased risk for 5-year mortality relative to patients with mild and moderate depressive symptom severity (HR = 3.708 [95%CI: 1.178-11.673], *p* = 0.025). After adjusting for patients’ age, gender, BI score and extent of tumor resection, adjuvant treatment, history of depression, tumor laterality, location and grade, severe depressive symptoms remained associated with elevated 5-year overall mortality risk of meningioma patients (HR = 7.083 [95% CI: 1.755 - 28.588], *p* = 0.006).

## DISCUSSION

Moderate to severe depressive and anxiety symptoms were diagnosed in 28% and 36% of brain tumor patients, respectively. Severe depressive symptoms were the most common in high-grade glioma patients, while anxiety symptom severity was similar between brain tumor histological diagnoses. In meningioma patients, greater depressive symptom severity was associated with shorter survival, and severe depressive symptoms predicted greater 5-year mortality risk adjusting for established clinical prognostic indicators. Anxiety and depressive symptoms were not associated with survival of high-grade and low-grade glioma patients.

In meningioma patient, survival was the shortest in patients with severe depressive symptoms, followed by patients with moderate and mild depressive symptoms, suggesting a dose-response type of association between depressive symptom severity and survival. After adjusting for demographic and clinical characteristics and depression histories, severe depressive symptoms remained associated with 7-fold increased risk of 5-year mortality of meningioma patients. The majority of previous studies investigating clinical significance of mental disorders and symptoms have focused on patients with high-grade gliomas [6]. To the best of our knowledge, this is the largest study to date investigating prognostic value of depressive/anxiety symptoms in meningioma patients. A study from Finland did not find an association of depressive symptoms with 5-year survival in 44 patients with various benign brain tumors that included 22 patients with meningiomas [13]. However, the association between depression and survival was not reported in a subgroup of meningioma patients. The association of greater depression symptom severity with worse prognosis of meningioma patients can be partially explained by lesser compliance with follow-up recommendation and by greater risk for unhealthy lifestyle behaviors, such as smoking and physical inactivity, in depressed patients [28]. Untreated depression can increase risk for cardiovascular and cerebrovascular disorders that become increasingly common with advancing age [29, 30]. Indeed, we found that nearly one third of deaths in meningioma patients were attributed to cerebrovascular and cardiovascular conditions. Studies evaluating behavioral consequences of depression in meningioma patients could potentially unveil mechanism underlying the observed association between depression and mortality. Meningioma patients can be cured and have normal life-expectancy following surgical tumor removal, therefore mental symptoms should not be ignored but should be appropriately addressed and managed.

Depressive symptoms were not associated with survival of high-grade and low-grade glioma patients. These findings are incongruent with previous reports showing that depressed glioma patients lived shorter relative to not-depressed patients [11, 12]. A retrospective study by Gathinji in 1055 high-grade glioma patients found that patients who were diagnosed with clinical depression by their primary care provider or psychiatrist before surgery lived shorter than patients without documented depression (median survival of 7 and 11 months, respectively) and this association was independent from patients’ age, functional status, tumor grade, extent of resection and adjuvant treatment [12]. However, due to retrospective study design (depression diagnoses were extracted from medical records) many depressed patients could have remained undiagnosed, since only 49 (5%) patients carried diagnosis of clinical depression pre-operatively. Indeed, it is well documented that depression often remains undiagnosed and not treated in brain tumor patients [11]. Another study in 598 glioma patients also reported that survival was shorter in depressed patients when compared to patients who were not depressed [11]. On the other hand, Mainio with colleagues did not find an association between prospectively evaluated depressive symptoms with five-year survival in a cohort of 15 high-grade glioma patients [13]. However, in the later study, survival was significantly shorter in depressed relative to not-depressed low-grade glioma patients and this association remained significant after adjusting for patient age, sex, and employment status [13]. Further methodologically rigorous studies investigating prognostic value of depression in glioma patients are needed taking into consideration established clinical (patients’ age and functional status), treatment-related (extent of resection and adjuvant therapies) and genetic prognostic indicators.

This was the first attempt to investigate prognostic role of pre-operative anxiety in brain tumor patients. Preoperative anxiety was not associated with survival of brain tumor patients. Others have linked pre-operative anxiety to greater morbidity and mortality after major cardiac surgery [31, 32]. However, studies in non-CNS cancer patients provided with mixed findings regarding an association of anxiety with survival [17–19]. These findings suggest that anxiety symptoms can be normal emotional reaction to cancer diagnosis and prognosis. Increase of anxiety symptom severity is expected before major surgical intervention. However, it should be noted that surgery-related anxiety was not specifically investigated, because we used the HADS-Anxiety subscale that was designed to evaluate anxiety symptom severity in hospital setting. Future studies should attempt to evaluate prognostic role of surgery-related pre-operative anxiety with outcomes of neurosurgical patients.

The proportion of patients with severe depressive symptoms was the greatest in high-grade glioma patients, while prevalence of anxiety symptoms was similar between brain tumor patients. Similar rates of elevated depression and anxiety symptoms were previously reported in brain tumor patients [6][14, 16]. For example, Pringle with colleagues in patients with primary and metastatic brain tumors, found that 49% of patients self-reported moderate to severe anxiety symptoms before surgery based on the HADS assessment results [14]. Another study in 72 primary and metastatic brain tumor patients found that 63% and 50% of patients had increased symptoms of state anxiety and trait anxiety, respectively, symptom severity before brain tumor surgery [16, 33]. Higher prevalence rates of preoperative anxiety in the latter two studies can be attributed to the fact that patients with primary and metastasis brain tumors were considered together. Greater anxiety is expected in patients with a known cancer diagnosis and with more advanced cancer, such as metastatic brain tumor. Similar distribution of elevated anxiety symptoms across brain tumor histological categories in our cohort suggests that anxiety is not related to brain tumor biology. Goebel and Mehdorn also found that anxiety symptom severity was similar between meningioma and high-grade glioma patients before and after neurosurgery as well as 6 months after surgery [8]. Increase of anxiety symptom severity before surgery can be attributed to surgery-related anxiety and fear of impeding diagnosis brain tumor diagnosis. Indeed, it was previously documented that anxiety symptom severity reduce after brain tumor surgery [26, 34].

It remains unclear if systematic screening for mental disorders should be performed in brain tumor patients as there are no studies showing clinical efficacy of such interventions [5, 35]. The HADS is the most commonly used self-rating scale for assessment of depressive symptom severity in neuro-oncology setting [6]. Brevity, good diagnostic properties and exclusion of somatic depressive symptoms are the major advantages of the HADS. Furthermore, the HADS allows simultaneous evaluation of anxiety symptom severity. Good psychometric properties (sensitivity of ≥ 80% and specificity of ≥88%) of the HADS for major depressive screening purposes in glioma patients was documented [36].

Our study has limitations. Lack of assessment for depressive and anxiety disorders is the major limitation of the present study. However, the HADS is commonly used and well validated diagnostic tool in brain tumor patients. Our cohort was heterogeneous in terms of histological diagnoses, and sample size of low-grade and high-grade glioma patients was small and therefore statistical power was low. Prospective studies elevating prognostic value of depression and anxiety in larger and homogenous cohorts of brain tumor patients are needed. Also, generalizability of our results is limited to patients undergoing brain tumor surgery and should not be extrapolated to outpatient setting because significant perioperative fluctuation of neuropsychological profile was previously documented [26, 34]. Furthermore, psychological state of brain tumor patients can depend on other symptoms imposed by brain tumor, such as level of consciousness, speech disturbances and focal neurological symptoms; therefore, further studies should investigate the importance of cognitive impairment for psychological distress symptoms severity and prognosis of brain tumor patients.

## CONCLUSIONS

Depressive and anxiety symptoms are common complications in glioma and meningioma patients. Depressive symptoms are more common in high-grade glioma patients while the prevalence of anxiety symptoms is not different across glioma and meningioma patients. Greater depressive symptom severity is associated with shorter survival and with greater five-year mortality risk of meningioma patients independently from clinical prognostic indicators. Depressive symptoms should be considered serious complication in meningioma patients and our findings suggest that antidepressant treatment should be considered for depressed meningioma patients. Further studies investigating prognostic significance of psychiatric co-morbidity in brain tumor patients are needed.
